# Analysis of endometrial thickness measured by transvaginal ultrasonography in obese patients

**DOI:** 10.1590/S1679-45082014AO2838

**Published:** 2014

**Authors:** Isabela Corrêa Barboza, Daniella de Batista Depes, Ilzo Vianna, Marisa Teresinha Patriarca, Raquel Martins Arruda, João Alfredo Martins, Reginaldo Guedes Coelho Lopes

**Affiliations:** 1 Hospital do Servidor Público Estadual, São Paulo, SP, Brazil.

**Keywords:** Obesity, Endometrium/anatomy & physiology, Body mass index

## Abstract

**Objective:**

To compare the endometrial echo values obtained by transvaginal ultrasonography with the body mass index of postmenopausal patients; to verify if there is higher prevalence of endometrial thickening in women with body mass index ≥30.

**Methods:**

This is an analytical and cross-sectional study that evaluated 294 patients. Postmenopausal women were included, and those on hormone therapy were excluded. The variables evaluated were body mass index (considering obesity if >30), number of pregnancies, years since menopause, and age. These variables were correlated with endometrial echography.

**Results:**

There was a statistically significant correlation between overweight and obese patients and increased endometrial thickness (p=0.0236). The correlation between age and endometrial echo was negative and statistically significant, that is, the older the woman, the lower the endometrial thickness (p=0.0478). Pregnancies and years since menopause showed no statistical significance in relation to endometrial echo, with p=0.0614 and p=0.115, respectively.

**Conclusion:**

There was positive and significant correlation between body mass index ≥30 and endometrial thickeness.

## RESUMO

**Objetivo:**

Relacionar os valores do eco endometrial obtidos à ultrassonografia transvaginal com o índice de massa corporal de pacientes na pós-menopausa; verificar se existe maior prevalência de espessamento endometrial naquelas com índice de massa corporal ≥30.

**Métodos:**

Trata-se de estudo analítico, transversal, que avaliou 294 pacientes. As pacientes incluídas estavam na pós-menopausa; foram excluídas aquelas que faziam uso de terapia hormonal. As variáveis avaliadas foram o índice de massa corporal, sendo considerado obesidade quando acima de 30, o número de gestações, o número de anos após a menopausa e a idade. Todas foram correlacionadas com os valores de eco endometrial.

**Resultados:**

Houve correlação estatisticamente significante entre as pacientes com sobrepeso/obesas e o aumento do eco endometrial (p=0,0236). A correlação entre idade e valores de eco endometrial foi negativa e significante, isto é, quanto maior a idade, menor o eco (p=0,0478). Gestações e anos após a menopausa não demonstraram significância com relação ao eco endometrial, com p=0,0614 e p=0,115, respectivamente.

**Conclusão:**

Houve correlação positiva e significante entre o aumento do eco endometrial e o índice de massa corporal ≥30.

## INTRODUCTION

The endometrium is a dynamic tissue that responds to variations in the levels of estrogen and progesterone, promoting proliferation, differentiation and desquamation.^(1)^

Biological and morphological changes may occur in the stroma and in endometrial glands, ranging from overproliferation to carcinoma.^(2,3)^ This may occur when the tissue is exposed to estrogen without the counterbalance of progesterone.^(1)^

This excessive exposure takes place especially in anovulatory cycles, polycystic ovary syndromes, estrogen secreting ovarian tumors, and obesity. Augmented fat tissue increases the action of the enzyme aromatase on androstenedione, thus increasing the quantity of stroma in the fat tissue. For this reason, obese patients are at greater risk of developing estrogen-dependent conditions.^(1,3,4)^

Obesity plays an important role in endometrial proliferation, having as its consequence endometrial hyperplasia with or without atypias. Giede et al. demonstrated that among patients with endometrial hyperplasia with atypia, 35.7% also presented endometrium carcinoma. According to the authors, the progression of atypical hyperplasia to carcinoma occurs in 23 to 25% of the cases.^(5–7)^ This fact is relevant because this is the seventh most frequent type of cancer among women, with, approximately, 290 thousand new cases per year in the world. The prevalence in developed countries is ten times greater than in developing countries. In Brazil, according to the *Instituto Nacional do Câncer* (INCA), an incidence of four new cases of endometrial hyperplasia per each 100,000 women was expected in 2012.^(8)^

The overexposure to estrogen in obese patients plays a fundamental role in endometrial alterations^(9)^, and this an important risk factor for endometrial cancer.^(10)^ In this manner, the detection of endometrial hyperplasia in its early stages is relevant in terms of public health.^(11)^

According to the World Health Organization (WHO), 12% of the world population is considered obese, and 3 million people die every year due to diseases associated to obesity - which is defined as abnormal or excessive accumulation of fat in the body.^(12)^

The most frequently used indicator to identify obese people is the body mass index (BMI), calculated as the individual´s weight (in kilograms) divided by the square of the height (in meters). Obesity is defined as BMI above 30. Values between 25 and 29.9 are considered overweight and from 18.5 to 24.9, eutrophic.^(10)^

The measurement of endometrial echography (EE) by ultrasound varies according to the phase of the woman's menstrual cycle. During desquamation the endometrial thickness varies from 0.5 to 7mm; in the proliferative phase, it may become as thick as 15mm, remaining stable in the second phase of the cycle. In post-menopause, EE is <5mm up to 8mm in patients on hormone therapy.^(13,14)^

## OBJECTIVE

To relate the values of the endometrial echography with the body mass index of postmenopausal patients and find if there is a greater prevalence of endometrial thickening in those with body mass index ≥30.

## METHODS

The study was submitted and approved by the Research Ethics Committee of the *Hospital do Servidor Público Estadual* “*Francisco Morato de Oliveira*”, in São Paulo (CAAE 08327613.6.0000.5463). Patients read and signed an Informed Consent Form.

It is an analytical, cross-sectional study that assessed 294 patients who received care in the gynecology outpatient clinic by the project called CARE (acronym in portuguese) of the *Hospital do Servidor Público Estadual de São Paulo* “*Francisco Morato de Oliveira*”. This project provides gynecological care and performs routine tests on a single day.

Data collection took place between May 15 and June 20, 2012. All patients included were postmenopausal. Those on hormone therapy were excluded. Some comorbidities, such as hypertension, diabetes and hypothyreoidism, were not considered exclusion criteria.

To calculate the BMI, all patients included in the study were weighed and measured by CARE's nursing staff, on the day of the office visit. Transvaginal ultrasound (TVUS) was done by the hospital's Radiology Service. In addition to BMI and EE, data collection also included age, date of last menses, and number of pregnancies.

Obesity was considered for BMI ≥30 and endometrial thickening was considered if above 5mm. All patients with this ultrasound abnormality had an outpatient hysteroscopy ordered. Data were tabulated using the software Microsoft Excel 2000 and tables were designed with the same program.

The statistical analysis correlated EE with age (Table 1), parity (Table 2), time since menopause (Table 3) and BMI. Analysis was done through correlation between EE and the previously mentioned variables.

**Table 1:** Ages divided into intervals and correlated with average endometrial echography, number of patients and percentages

Age	Average of EE values	n (%)
40–45	3.75	4 (1.4)
46–50	3.10	10 (3.4)
51–55	3.04	60 (20.4)
56–60	3.37	58 (19.7)
61–65	2.85	70 (23.8)
66–70	2.81	41 (13.9)
71–75	2.43	20 (6.8)
76–80	3.76	15 (5.1)
80–90	2.88	16 (5.4)

EE: endometrial echography

**Table 2:** Correlation between parity, endometrial echography mean for each interval, number of patients in each interval and percentages

Parity	Average of EE values	n (%)
0	2.53	35 (11.8)
1	2.60	24 (8.1)
2–3	3.19	139 (47.3)
<3	3.38	96 (32.6)

EE: endometrial echography.

**Table 3:** Years since menopause divided into intervals and correlated with endometrial echography mean, number of patients and percentages

Years since menopause	Average of EE values	n (%)
1–5	3.11	53 (18)
6–10	3.36	73 (24.7)
11–15	2.88	64 (21.7)
16–20	2.71	41 (13.9)
>20	2.96	63 (21.4)

EE: endometrial echography.

Non-parametric Kruskal-Wallis, Spierman and Duun tests were applied, and statistical significance was p<0.05.

## RESULTS

Nine out of 294 patients analyzed had endometrial thickening diagnosed. This is equivalent to a frequency of 3.06%. The mean EE was 3.04mm and average age was 62.3 years (approximately 62 years and 4 months) for all patients. With regard to the number of pregnancies, the most prevalent group was that of multigravidas (second and third gestation), accounting for 50.2%. The mean time after menopause was 11.3 years, and average BMI was 28.67 (Table 4).

**Table 4:** Mean values and variables

Variable	Mean
EE (mm)	3.04
Pregnancies, number	Multigravida (second, third gestation)
Age (years)	62.3
BMI	28.67
Years since menopause	11.3

EE: endometrial echography; BMI: body mass index.

With respect to BMI, 73.8% of patients presented an index >25, in which 108 (36.73%) had a BMI between 25 and 29.9, and 109 (37.7%) >30.

There was a negative correlation between age and EE, demonstrating that the older the woman, the smaller the EE, with statistical significance, p=0.048. As to years since menopause, there was no difference in relation to EE, with p=0.115. The number of pregnancies also did not present a significant relation with EE, with p=0.614. There was positive and significant correlation between BMI and EE, demonstrating that the greater the BMI, the greater the EE, with p=0.0236.

## DISCUSSION

With regard to age and EE, the statistical analysis demonstrated that there is a negative correlation between these variables, that is, the greater the age, the smaller the EE (p=0.0478). In postmenopause, the endometrium is atrophic.^(15)^ Although expected, there are no articles in the literature relating age with EE reduction, as reported in the present study. Other authors say that aging is not an isolated risk factor for endometrial disease. Most times, there was an association with underlying diseases, such as diabetes, hypertension and obesity.^(8,10,15)^

We did not find statistical significance relating years since menopause with EE values (p=0.612). However, Dossus et al. wrote that postmenopausal patients after 50.9 years of age present greater risk for endometrial cancer when compared to 49.8 year-old patients.

As to the number of pregnancies, parity and EE values, Takeda et al. ^(1)^ reported that patients with an early menarche, late menopause, and nulliparity are more exposed to estrogen during lifetime, presenting greater risk of endometrial diseases. Dossus et al.^(10)^ also mentioned nulliparity as a risk factor for endometrial cancer, but they do not mention this variable as a risk for endometrial thickening. Differently from Epplen et al.^(2)^, who indicated nulliparity and obesity as risk factors for endometrial hyperplasia, with or without atypias. The present study did not find statistical significance between the number of pregnancies and endometrial thickening (p=0.0614).

The literature indicates that obese patients present greater frequency of endometrial thickening as compared to eutrophic women,^(2,5,6,9)^ a statement which this study confirmed.

There was a significant correlation between increase in BMI and EE (p=0.0256), but what drew our attention was that there was no difference between overweight (BMI>25) and obese (BMI>30) patients in relation to EE increase. That is, it demonstrated that there is no difference between overweight and obesity in relation to EE. Canchola et al.^(16)^ and Charneco et al.^(17)^ demonstrated that the risk for endometrial disease in overweight women is twice greater than in those with BMI <25. Obese people, in contrast, presented a four-fold greater risk.

Among the 294 patients analyzed, 9 presented endometrial thickening - 3 eutrophic, 3 overweight and 3 obese. In this manner, 66.67% of EE alterations were in the risk group (overweight and obese).

It's necessary to comment about the profile of the patients analyzed. Of the total, 217 (73.8%) presented BMI >25 and only 77 were considered eutrophic. These numbers are worrying, considering that obesity is a risk factor not only for endometrial diseases, but also for diabetes and cardiovascular diseases, such as hypertension and thromboembolic phenomena. This group of diseases has greater mortality rates than endometrial cancer and account for the death of 8.5 million women every year (one third of all deaths in women). Cardiovascular disease is the leading cause of death among women worldwide, according to the American Heart Association (AHA)*.* ^(18)^

## CONCLUSION

There was a positive and significant correlation between increase in endometrial echography and body mass index, demonstrating the influence of obesity on endometrial thickening.

## References

[1] Takeda G, Lopes RG, Lopes RG (2011). Hiperplasia de endométrio. O endométrio.

[2] Epplein M, Reed SD, Voigt LF, Neuton KM, Holt VL, Weiss NS (2008). Risk of complex and atypical hyperplasia in relation to anthropometric measures and reproductive history. Am J Epidemiol.

[3] Banzato PC, Lopes RG, Lopes RG (2011). A cavidade uterina e o endométrio nas diferentes fases do ciclo menstrual. O endométrio.

[4] Sedar Serin I, Ozçelik B, Basbug M, Ozsahim O, Yilmazsoy A, Erez R (2003). Effects of hypertension and obesity on endometrial thickness. Eur J Obstet Gynecol Reprod Biol.

[5] Viola AS, Gouveia D, Andrade L, Aldrighi JM, Viola CF, Bahamondes L (2008). Prevalence of endometrial cancer and hyperplasia in non-symptomatic overweight and obese women. Aust N Z J Obstetic Gynaecol.

[6] Heller DS, Mosquera C, Goldsmith LT, Cracchiolo B (2011). Body mass index of patients with endometrial hyperplasia: comparison to patients with proliferative endometrium and abnormal bleeding. J Reprod Med.

[7] Gied KC, Yen TW, Chibbar R, Pierson RA (2008). Significance of concurrent endometrial cancer in women with a preoperative diagnosis of atypial endometrial hyperplasia. J Obstet Gynaecol Can.

[8] Instituto Nacional de Câncer (INCA) Estimativa de câncer no Brasil em 2012 [Internet].

[9] Lu L, Risch H, Irwin ML, Mayne ST, Cartmel B, Schwartz P (2011). Long-term overweight and weight gain in early adulthood in association with risk of endometrial cancer. Int J Cancer.

[10] Dossus L, Rinaldi S, Becker S, Lukanova A, Tjonneland A, Olsen A (2010). Obesity, inflammatory markers, and endometrial cancer risk: a prospective case-control study. Endocr Relat Cancer.

[11] Park SL, Goodman MT, Zhang ZF, Kolonel LN, Henderson BE, Setiawan VW (2010). Body size, adult BMI gain and endometrial cancer risk: the multiethnic cohort. Int J Cancer.

[12] Associação Brasileira para Estudo da Obesidade e Síndrome Metabólica (ABESO). Organização Mundial de Saúde (OMS) (2012). Relatório: obesidade mata mais de 2,8 milhões de pessoas ao ano [Internet].

[13] Marceline C, Petti DA, Lopes RG (2011). Embriologia e anatomia do endométrio. O endométrio.

[14] Nutis M, García KM, Nuwayhid B, Mulla Z, ElMasri W (2008). Use of ultrasonography cut point for diagnosis endometrial pathology in postmenopausal women with multiple risk factors for endometrial cancer. J Reprod Med.

[15] Deppes DB, Lopes RG (2011). O endométrio na pós-menopausa. O endométrio.

[16] Canchola AJ, Chang ET, Bernstein L, Largent JA, Reynolds P, Deapen D (2010). Body size and the risk of endometrial cancer by hormone therapy use in postmenopausal women in the California Teachers Study control. Cancer Causes Control.

[17] Charneco E, Ortiz AP, Venegas-Ríos HL, Romaguera J, Umpierre S (2010). Clinic-based-case-control study of the association between body mass index and endometrial cancer in Puerto Rican women. P R Health Sci J.

[18] American Heart Association (AHA) Obesidade e sobrepeso: American Heart Association [Internet].

